# A92 FEMALE AUTHORSHIP IN GASTROENTEROLOGY RANDOMIZED CONTROL TRIALS: 2011 - 2021

**DOI:** 10.1093/jcag/gwac036.092

**Published:** 2023-03-07

**Authors:** C S Liu, Z X Lin, K Kroeker

**Affiliations:** 1 Department of Medicine, University of Alberta Faculty of Medicine and Dentistry; 2 Department of Cell Biology, University of Alberta Faculty of Science, Edmonton, Canada

## Abstract

**Background:**

Although tremendous strides have been made in the participation of women in medicine, female continues to be underrepresented in leadership positions and higher-level academic medicine. An important factor in determining career advancement in academic medicine is the quality and quantity of an individual’s scholarly publications. To date, no study has looked at female authorship in gastroenterology (GI) randomized control trials (RCTs), which remains the gold standard for evaluating intervention effectiveness.

**Purpose:**

The primary outcome is to assess female authorship in gastroenterology randomized control trials from 2011 to 2021, and the secondary outcome is to assess female authorship within GI subspecialty RCT publications.

**Method:**

In this observational study, the gender of the first and last author of gastroenterology RCTs from January 1, 2011 to December 31, 2021 was assessed. Python (v3.8.12) was used to extract publication data from PubMed. A validated algorithm, genderize.io, was used to determine gender. Author first names that cannot be determined by the algorithm were manually searched on publicly-available profiles.

**Result(s):**

A total of 5690 original gastroenterology RCTs were included from January 1, 2011 to December 31, 2021. The gender of the first and senior authors of the papers was determined for 5668 (99.6%) first authors and 5656 (99.4%) senior authors. Overall, 1937 (34.1%) of the first authors and 1138 (20.0%) of senior authors were female.

There was an increase in the proportion of female first authors over the past decade, from 25.4% in 2011 to 37.8% in 2021 (p<0.05). For senior authors, there was a more gradual increase in female authorship from 14.2% in 2011 to 21.6% in 2021 (p<0.05). (Figure 1)

Within GI subspecialties, 612 RCTs were included for inflammatory bowel disease, 1143 RCTs were included for hepatology, and 1856 RCTs were included for therapeutic endoscopy from January 1, 2011 to December 31, 2021. Further analysis will be performed to determine the gender trend for GI subspecialties.

**Image:**

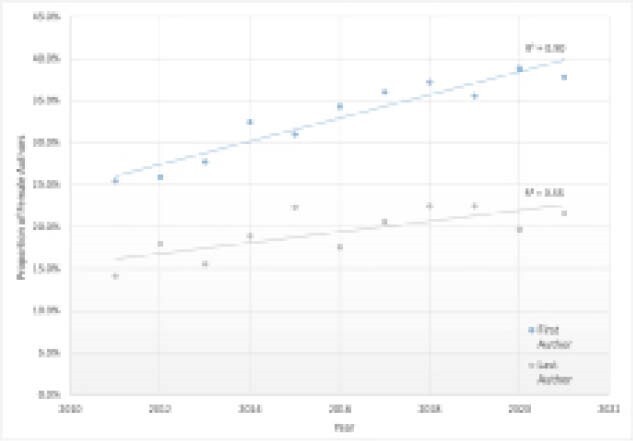

**Conclusion(s):**

Female authorship in gastroenterology RCTs has increased from 2011 to 2021, although the rate of senior authorship has increased to a slower extent compared to first authors. Across all years, female authorship in gastroenterology RCTs has been lower than males.

**Please acknowledge all funding agencies by checking the applicable boxes below:**

None

**Disclosure of Interest:**

None Declared

